# Reliability of Telepsychiatry Assessments Using the Attention-Deficit/Hyperactivity Disorder Rating Scale-IV for Children With Neurodevelopmental Disorders and Their Caregivers: Randomized Feasibility Study

**DOI:** 10.2196/51749

**Published:** 2024-02-19

**Authors:** Shunya Kurokawa, Kensuke Nomura, Nana Hosogane, Takashi Nagasawa, Yuko Kawade, Yu Matsumoto, Shuichi Morinaga, Yuriko Kaise, Ayana Higuchi, Akiko Goto, Naoko Inada, Masaki Kodaira, Taishiro Kishimoto

**Affiliations:** 1 Department of Neuropsychiatry, School of Medicine Keio University Tokyo Japan; 2 Department of Child Psychiatry Shimada Ryoiku Medical Center for Challenged Children Tokyo Japan; 3 Department of Child and Adolescent Mental Health Aiiku Clinic Tokyo Japan; 4 Department of Child and Adolescent Psychiatry Tokyo Metropolitan Children's Medical Center Tokyo Japan; 5 Tsurugaoka Garden Hospital Tokyo Japan; 6 Hiratsuka City Hospital Kanagawa Japan; 7 Department of Clinical Psychology Taisho University Tokyo Japan; 8 Hills Joint Research Laboratory for Future Preventive Medicine and Wellness Keio University School of Medicine Tokyo Japan

**Keywords:** acceptability, ADHD, application, attention-deficit/hyperactivity disorder, autism spectrum disorders, autism, child, children, diagnosis, management, neurodevelopmental disorder, neurodevelopmental, psychiatrists, reliability, telepsychitatry

## Abstract

**Background:**

Given the global shortage of child psychiatrists and barriers to specialized care, remote assessment is a promising alternative for diagnosing and managing attention-deficit/hyperactivity disorder (ADHD). However, only a few studies have validated the accuracy and acceptability of these remote methods.

**Objective:**

This study aimed to test the agreement between remote and face-to-face assessments.

**Methods:**

Patients aged between 6 and 17 years with confirmed *Diagnostic and Statistical Manual of Mental Disorders, Fifth Edition* diagnoses of ADHD or autism spectrum disorder (ASD) were recruited from multiple institutions. In a randomized order, participants underwent 2 evaluations, face-to-face and remotely, with distinct evaluators administering the ADHD Rating Scale-IV (ADHD-RS-IV). Intraclass correlation coefficient (ICC) was used to assess the reliability of face-to-face and remote assessments.

**Results:**

The participants included 74 Japanese children aged between 6 and 16 years who were primarily diagnosed with ADHD (43/74, 58%) or ASD (31/74, 42%). A total of 22 (30%) children were diagnosed with both conditions. The ADHD-RS-IV ICCs between face-to-face and remote assessments showed “substantial” agreement in the total ADHD-RS-IV score (ICC=0.769, 95% CI 0.654-0.849; *P*<.001) according to the Landis and Koch criteria. The ICC in patients with ADHD showed “almost perfect” agreement (ICC=0.816, 95% CI 0.683-0.897; *P*<.001), whereas in patients with ASD, it showed “substantial” agreement (ICC=0.674, 95% CI 0.420-0.831; *P*<.001), indicating the high reliability of both methods across both conditions.

**Conclusions:**

Our study validated the feasibility and reliability of remote ADHD testing, which has potential benefits such as reduced hospital visits and time-saving effects. Our results highlight the potential of telemedicine in resource-limited areas, clinical trials, and treatment evaluations, necessitating further studies to explore its broader application.

**Trial Registration:**

UMIN Clinical Trials Registry UMIN000039860; http://tinyurl.com/yp34x6kh

## Introduction

Attention-deficit/hyperactivity disorder (ADHD) and autism spectrum disorder (ASD) are caused by genetic and environmental factors. Children with ADHD or ASD are recognized to be at risk for emotional and behavioral difficulties during adolescence, young adulthood, and beyond, owing to social adjustment difficulties. Hence, early detection and intervention are crucial to prevent the development of additional comorbidities [1-3].

The most recent US epidemiological study reported a prevalence of 9.5% for ADHD and 2.5% for ASD [4], making ADHD the most common neurodevelopmental disorder. In Japan, although epidemiological studies on ADHD in children are lacking, the prevalence rate in adults has been reported to be 1.65% [5]. However, children with ADHD or ASD face many obstacles in accessing specialized medical care. One of the biggest challenges is the global shortage of child psychiatrists, leading to long waiting times for diagnosis. For example, in Canada and the United States, the average wait times are 7 and 13 months, respectively [6,7]. This is particularly concerning in rural or isolated areas without access to specialized medical facilities.

In addition, these children may have difficulties leaving their homes and engaging in social interactions (ie, social withdrawal). Even when they have access to specialized medical facilities, some may find it extremely difficult to see a doctor in person. Moreover, it has been reported that these children have trouble with the time management associated with ADHD, resulting in high dropout rates during treatment [8,9].

Moreover, accurate diagnosis requires close observation by medical staff and a thorough interview with caregivers regarding the child’s developmental history [10,11]. However, a lack of trained evaluators can lead to difficulty in accurate assessment. Children’s evaluations are susceptible to being influenced by the halo effect (the distortion of evaluations about other characteristics due to the salient features they possess) and the contrast effect (a greater perception of difference than the actual difference) [12,13].

One possible approach to solving these problems is remote diagnosis and evaluation. In recent years, because a large part of psychiatric treatment and assessment has consisted of conversations with patients, remote evaluation and treatment using remote digital tools can be effective, particularly in clinical trials.

Therefore, the US Food and Drug Administration and the European Medicines Agency are promoting remote central evaluation using videoconferencing systems in psychiatric trials [14] and physician-led clinical trials [15], which are becoming increasingly common. To remotely perform a severity diagnosis in clinical trials, it is necessary to verify the degree of agreement with the usual face-to-face assessment.

Only a few studies have validated the agreement of remote assessment tests for developmental disorders with the usual face-to-face assessment [16-18]. A study reported that the Autism Diagnostic Interview–Revised Edition score from face-to-face interviews with caregivers of 20 children with ASD was equivalent to the Autism Diagnostic Interview–Revised Edition score from telephone interviews (*r*=0.73-0.90) [16]. The Autism Diagnostic Observation Schedule–2, considered the gold standard for ASD observation ratings, was administered to 23 adults with ASD and showed an intraclass correlation coefficient (ICC) of 0.92 between face-to-face and remote assessments [17], with the limitation that the ratings were made by the same rater. One study used a mobile app to video record scenes in which parents were concerned about their child’s development at home [18]. Among the 40 children with ASD and 11 children with typical development, 88.2% of diagnoses agreed with the face-to-face diagnosis using the Autism Diagnostic Observation Schedule and other tools (κ=0.75; sensitivity=84.9%; specificity=94.4%).

However, research on the remote assessment of ADHD ratings is limited. The ADHD Rating Scale-IV (ADHD-RS-IV) [19,20] is the gold standard tool used to support the diagnosis and severity assessment of ADHD, and it has been used in practice in many clinical trials [21,22]. It has been reported that the sensitivity and specificity of ADHD-RS-IV in Japan above the 90th percentile were 89.13% and 94.07%, respectively [23]. This study aimed to test whether the remote method is equivalent to the face-to-face method for children with ADHD and ASD and their caregivers by administering ADHD-RS-IV and determining the ICC and to verify the validity and feasibility of remote assessment.

## Methods

### Participants

Patients were recruited at Keio University Hospital and 4 collaborating institutions (Shimada Ryoiku Medical Center for Challenged Children, Aiiku Clinic, Tokyo Metropolitan Children’s Medical Center, and Tsurugaoka Garden Hospital). The inclusion criteria were as follows: (1) confirmation of *Diagnostic and Statistical Manual of Mental Disorders, Fifth Edition* (DSM-5) [24] diagnoses of ADHD or ASD; (2) aged between 6 and 17 years at the time of obtaining consent; and (3) if receiving pharmacotherapy, the treatment was stable for at least 3 months before obtaining consent.

The exclusion criteria were as follows: (1) either the child or caregiver had a hearing or visual impairment that made it difficult to use remote tools, even with corrective devices, such as glasses or hearing aids; (2) there were no caregivers with information related to the participants’ early childhood; (3) individuals had comorbid symptoms, such as hallucinations and delusions, which made it challenging to engage in the study, as determined by the clinician; and (4) there were plans to initiate new treatments, such as pharmacotherapy or psychotherapy, during the observation period.

### Baseline Assessments

Background information and data, such as age, sex, diagnosis, medication, duration of illness, and intelligence test results, were collected from medical records. For the baseline evaluation, the Autism-Spectrum Quotient–Japanese version for children, the Conners 3 Japanese version to evaluate ADHD symptoms by caregivers, the Strengths and Difficulties Questionnaire to assess social adaptation, and the Short Sensory Profile to assess sensory characteristics were administered.

### Study Procedure

The ADHD-RS-IV and Childhood Autism Rating Scale–2 scores were used as primary outcomes to compare face-to-face and remote developmental evaluations.

After the baseline assessments, the participants underwent evaluations twice, either face-to-face or remotely. Recall bias could have occurred if the same evaluator had conducted both assessments. Therefore, the order of the face-to-face and remote evaluations was randomized for each participant, and different evaluators conducted the evaluations. To reduce the burden on the participants, minimize the practice effect, and avoid temporal changes, the interval between face-to-face and remote evaluations was set at least 2 weeks and no more than 3 months apart.

After completing the 2 assessments, the participants were given a questionnaire to report their satisfaction with the remote assessment. Additionally, given that previous studies showed long waiting periods for accessing child psychiatry specialists, we gathered background information on the waiting period at a specialist’s initial meeting. We also considered cases where specialized hospitals and clinics were not within one’s living area and collected data on the average travel time for outpatient visits and waiting time for their usual visit.

The 3 evaluators were licensed psychologists in developmental testing who had undergone sufficient training and had confirmed their agreement rates before conducting the evaluations. Evaluators rotated as needed for both in-person and remote assessments, rather than having specific evaluators for each. None of them was involved in the participants’ assessments or treatment during their usual visits.

As preliminary training before starting the research, 3 examiners administered the ADHD-RS-IV to 5 patients.

The remote smartphone assessment tool “Curon” by MICIN Co Ltd was used for the telemedicine evaluation. The participants were seated in front of a smartphone in their homes and were introduced to a remote evaluator. Remote evaluators administered their evaluations in a room at the Keio University Hospital using a PC. Although it was assumed that a download and upload environment of 50 Mbps or higher would be perfect for remote video applications, stable communication was achieved at approximately 5 Mbps, which, in most cases, fulfilled the Japanese standard 4G network. The assessments began after confirming that there were no interruptions in the video or audio environment.

### Statistical Analysis

Test-retest reliability was assessed using the ICC for continuous variables, with the following standard performance parameters: almost perfect (0.81-1.00); substantial (0.61-0.80); moderate (0.41-0.60); fair (0.21-0.40); and slight (0.0-0.20) [25]. The ICC within the same rater when testing the same patient multiple times was assumed to be 0.90. Assuming that the ICC between the remote and face-to-face tests was 0.8, and to achieve a 95% CI width of 0.3 with a probability of >80%, a sample size of 31 was required. It was also assumed that the analysis would be conducted by dividing the participants into 2 groups: ASD and ADHD. Therefore, the required sample size was set at 62 [26]. Normally distributed data are described as mean (SD). Categorical variables are presented as numbers and percentages. All variables were inspected using histograms, q-q plots, and Kolmogorov-Smirnov tests before statistical analyses were conducted to test the distribution. All analyses were 2-sided with an α value of .05. Statistical analyses were conducted using the SPSS software (version 25.0; SPSS Inc).

### Ethical Considerations

The study protocol was approved by the ethics committee of Keio University School of Medicine (20190301). The study was registered in the UMIN Clinical Trial Registry (UMIN000039860). Informed consent was obtained from all caregivers. For the children, an informed assent form was used, tailored to their age and understanding, and written confirmation of their consent was obtained after careful explanation. The personal information collected in this study was securely stored in a locked cabinet located within a lockable room belonging to the corresponding author. The information obtained through this study was anonymized by removing personal identifiers and assigning research numbers at each institution and then was stringently managed and preserved under the supervision of the corresponding author. In the event of an exacerbation of psychiatric symptoms as a result of this study, appropriate treatment was to be administered. No financial compensation was provided.

## Results

### Participants

The demographic characteristics of the participants are shown in Table 1. A total of 75 patients consented to participate in the study. Overall, 2 participants were from Keio University Hospital, 44 were from Shimada Ryoiku Medical Center for Challenged Children, 13 were from Aiiku Clinic, 10 were from Tokyo Metropolitan Children’s Medical Center, and 6 were from Tsurugaoka Garden Hospital. Of these, 1 participant from Shimada Ryoiku Medical Center for Challenged Children dropped out owing to time limitation. Among the 74 remaining participants, 17 (23%) were girls and 57 (77%) were boys. All patients were Asian (Japanese). Their ages ranged from 6 to 16 years, with an average of 10.4 (SD 2.5) years. The primary clinical diagnoses were as follows: 31 (42%) individuals were diagnosed with ASD, 43 (58%) were diagnosed with ADHD, and 22 (30%) had comorbid ASD and ADHD. Of the 61 caregivers, 47 (77%) had previous experience with remote video calls, whereas 14 (23%) did not.

**Table 1:** Demographic characteristics.

Characteristics	All patients (n=74)	Patients with ADHD^a^ (n=43)	Patients with ASD^b^ (n=31)
Sex (female), n (%)	17 (16)	12 (28)	5 (16)
Age (years), mean (SD)	10.4 (2.5)	10.5 (2.4)	10.1 (2.5)
SDQ^c^, mean (SD)	21.94 (5.31)	21.09 (5.35)	22.93 (5.16)
AQ^d^, mean (SD)	24.68 (7.38)	22.86 (6.72)	27.23 (7.62)
Conners 3, mean (SD)	112.84 (38.22)	108.34 (39.94)	118.53 (35.77)
SSP^e^, mean (SD)	70.78 (20.26)	67.44 (20.38)	75.19 (19.55)
CARS-2^f^ (face-to-face), mean (SD)	35.97 (4.90)	34.19 (4.49)	38.37 (4.43)
ADHD-RS-IV^g^ total (face-to-face), mean (SD)	27.25 (9.88)	26.88 (10.38)	27.77 (9.29)
ADHD-RS-IV hyperactivity/impulsiveness (face-to-face), mean (SD)	8.08 (6.08)	7.95 (6.60)	8.27 (5.39)
ADHD-RS-IV total inattention (face-to-face), mean (SD)	19.17 (5.29)	18.93 (5.28)	19.50 (5.37)

^a^ADHD: attention-deficit/hyperactivity disorder.

^b^ASD: autism spectrum disorder.

^c^SDQ: Strength and Difficulties Questionnaire.

^d^AQ: Autism-Spectrum Quotient.

^e^SSP: Short Sensory Profile.

^f^CARS-2: Childhood Autism Rating Scale-2.

^g^ADHD-RS-IV, Attention-deficit/Hyperactivity Disorder Rating Scale-IV.

Participants were asked how long the waiting period was until the first visit for a child psychiatrist appointment, and the average was found to be 79.0 (SD 57.1; range 1-200) days. Regarding their usual visits, it took 36.8 (SD 20.7; range 5-100) minutes from leaving home to arriving at the hospital. The average waiting time for a usual appointment was 24.0 (SD 14.6; range 5-60) minutes. The potential time that could be saved using remote visits (by adding visiting time × 2 and waiting time) was 97.7 (SD 42.5; range 40-260) minutes.

### ICCs of ADHD-RS-IV Between Face-to-Face and Remote Assessments

The ICCs obtained after preliminary training were as follows: 0.987 (95% CI 0.934-0.999; *P*<.001) for the total score; 0.991 (95% CI 0.957-0.999; *P*<.001) for the hyperactivity/impulsivity subscore; and 0.807 (95% CI 0.022-0.978; *P*=.02) for the inattention subscore.

**Figure 1:**
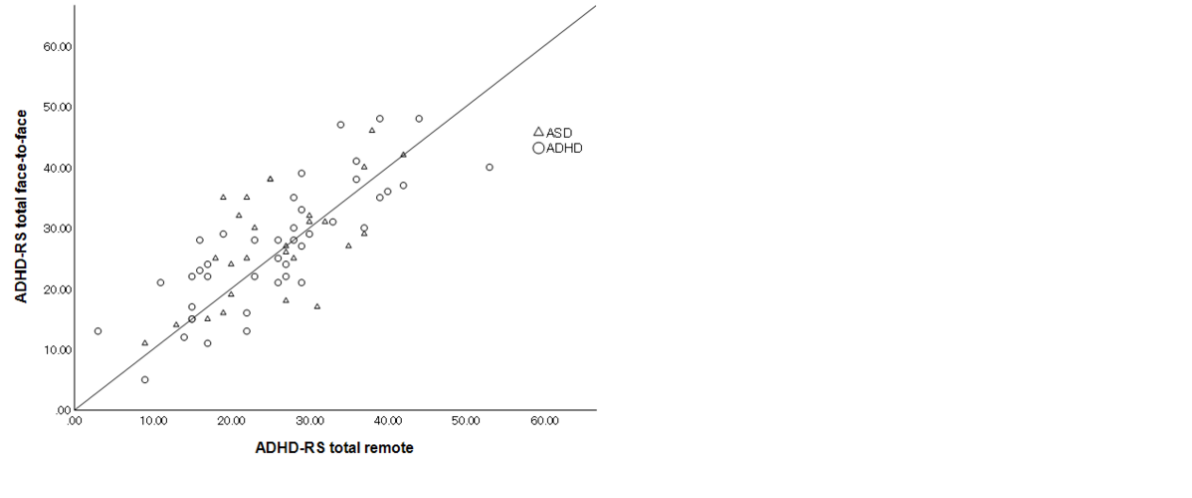
Intraclass correlations of total Attention-Deficit/Hyperactivity Disorder Rating Scale-IV (ADHD-RS-IV) score between face-to-face and remote assessments in all participants (autism spectrum disorder [ASD] and attention-deficit/hyperactivity disorder [ADHD]).

**Figure 2:**
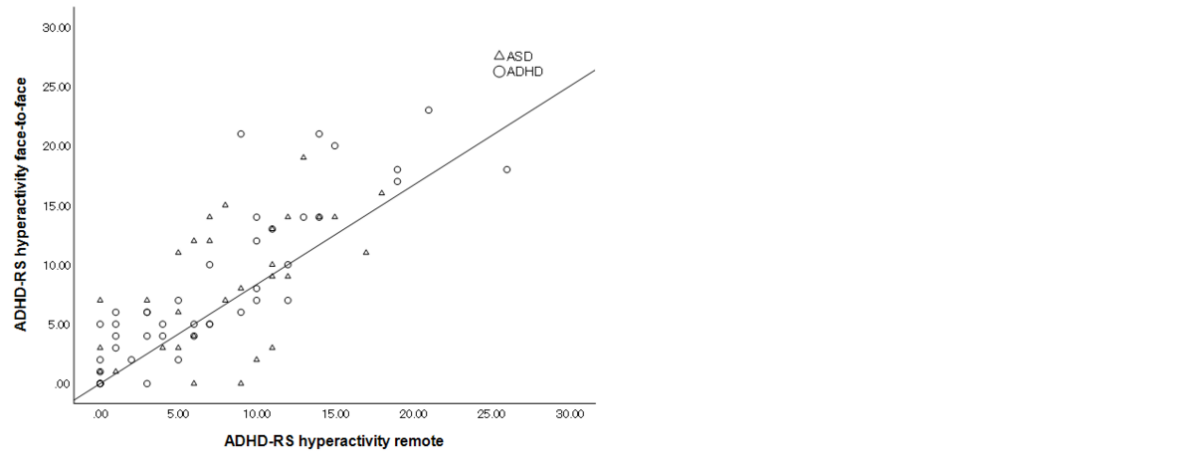
Intraclass correlations of Attention-Deficit/Hyperactivity Disorder Rating Scale-IV (ADHD-RS-IV) hyperactivity/impulsiveness subscore between face-to-face and remote assessments in all participants (autism spectrum disorder [ASD] and attention-deficit/hyperactivity disorder [ADHD]).

**Figure 3:**
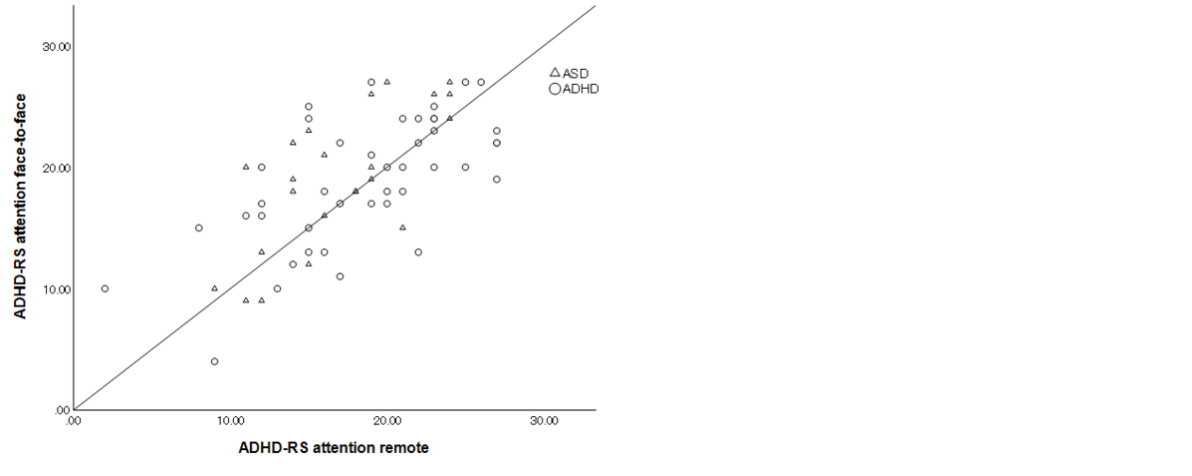
Intraclass correlations of Attention-Deficit/Hyperactivity Disorder Rating Scale-IV (ADHD-RS-IV) inattention subscore between face-to-face and remote assessments in all participants (autism spectrum disorder [ASD] and attention-deficit/hyperactivity disorder [ADHD]).

The ICCs are shown in Figures 1-3. The ICC of the total ADHD-RS-IV was 0.769 (95% CI 0.654-0.849; *P*<.001), which was “substantial,” according to the Landis and Koch criteria [25]. The ICC of the ADHD-RS-IV hyperactivity/impulsiveness and inattention subscores were 0.779 (95% CI 0.669-0.856; *P*<.001) and 0.667 (95% CI 0.515-0.778; *P*<.001), respectively, indicating “substantial” agreement. In patients with ADHD as their primary diagnosis, the ICC for total score was 0.816 (95% CI 0.683-0.897; *P*<.001), indicating “almost perfect” agreement; the ICC for hyperactivity/impulsiveness was 0.861 (95% CI 0.756-0.923; *P*<.001), indicating “almost perfect” agreement; and the ICC for inattention score was 0.642 (95% CI 0.423-0.790; *P*<.001), indicating “substantial” agreement. In patients with ASD as their primary diagnosis, the ICC for total score was 0.674 (95% CI 0.420-0.831; *P*<.001), indicating “substantial” agreement; the ICC for hyperactivity/impulsiveness was 0.591 (95% CI 0.299-0.782; *P*<.001), indicating “moderate” agreement; and ICC for inattention score was 0.733 (95% CI 0.511-0.863; *P*<.001), indicating “substantial” agreement.

There were no clear sex differences in the ICCs. In regard to age, comparing the older age group (≥11 years; n=37) and the younger age group (≤10 years; n=37), the ICC for total score was 0.675 (95% CI 0.464-0.814; *P*<.001) in the younger age group and 0.757 (95% CI 0.558-0.873; *P*<.001) in the older age group. The ICC for hyperactivity/impulsiveness was 0.706 (95% CI 0.509-0.833; *P*<.001) in the younger age group and 0.730 (95% CI 0.515-0.858; *P*<.001) in the older age group. The ICC for inattention score was 0.599 (95% CI 0.357-0.766; *P*<.001) in the younger age group and 0.659 (95% CI 0.408-0.818; *P*<.001) in the older age group.

## Discussion

### Principal Findings

This is the largest study to date on the validation and feasibility of remote ADHD testing. We found good agreement between the remote- and face-to-face–administered ADHD-RS-IV. This study also showed significant potential benefits for children and their caregivers in terms of reducing hospital visits and waiting times.

Additionally, as many as 14 (23%) out of the 61 caregivers who participated in the study had no previous experience with remote video calls; however, this did not pose a significant problem. This can be partly attributed to the staff providing detailed instructions. However, it is also possible that children who had attended internet-based classes at school because of the COVID-19 pandemic were able to provide guidance on the operation of the technology.

The results showed that the agreement rate between the face-to-face and remote assessments was lower in patients with ASD than in those with ADHD, according to the ADHD-RS-IV. One possible reason for the difference in results between patients with ADHD and ASD is the variability in interpretation among caregivers and evaluators. For example, when asked, “Does it seem like the child is not listening when spoken to?” caregivers of children with ASD, who may already have limited social interaction, may readily answer “yes” or “always.” In contrast, they may consider it a symptom of autism rather than inattentiveness related to ADHD and answer “no.” The wide range of interpretations provided by caregivers and evaluators may be a contributing factor [27].

The ADHD-RS-IV results indicated that the inattention ICC scores were numerically lower than the hyperactivity scores. This may be due to the remote assessment environment. At the time of enrollment, a quiet environment was recommended for remote assessments. However, because of the space and density of patients’ homes, situations often arose where there were numerous stimuli, such as the presence of toys or the assessment interview being conducted while other siblings were nearby or in the same room. These circumstances, compared with a controlled hospital consultation room, may have influenced the raters’ impressions of the participants in remote assessments. Moreover, when assessments take place at home, caregivers may feel uncomfortable explaining the severe or negative condition of their children in the presence of their child or other family members. It would be desirable to confirm in advance whether a space can be secured or a time when no other siblings are present can be arranged for remote assessment.

Our results have shown higher agreement rates in the older age group (≥11 years) compared to the younger age group (≤10 years). Symptoms such as hyperactivity and inattention in younger children may be more apparent in their home environment than in controlled settings due to familiarity and less stimulation control, resulting in a slight decrease in agreement with the assessment. Although there may be a benefit to internet-based assessment in regard to observing how the child typically behaves in relaxed homes, older children may be better suited for remote assessment in terms of agreement with face-to-face evaluation.

The potential time-saving effect, considering the time for visits to the hospital and waiting time at the hospital, was found to be 97.7 (SD 42.5; range 40-260) minutes. Given the increase in dual-income households in Japan, which has resulted in less time being spent with children, it would be highly meaningful if we could save this amount of time using remote assessments without reducing quality.

In addition, this research was conducted during the COVID-19 pandemic (the state of emergency was first declared on March 13, 2020, and was removed from the special measures law on May 8, 2023), which may have influenced the results. Although telemedicine was introduced in Japan, it was not widely adopted because of reluctance from the perspective of medical fees. Therefore, in many cases, people had to go to the hospital, facing the risk and anxiety of infection. The importance of having such tools ready to prepare for future outbreaks cannot be overlooked. While the demand for and evidence of telemedicine are expanding, there are also challenges. Issues such as the digital divide, which refers to the disparity that arises between people who can use the internet and computers and those who cannot, and in Japan specifically, the difficulty in widespread adoption due to regulations preventing billing for such services, are notable.

Based on the results of our study, telemedicine may be used under the following conditions: (1) in areas where there is a shortage of medical resources, such as public health and developmental support centers, by collaborating with child psychiatrists, clinical psychologists, and other medical professionals; (2) in central evaluations in clinical trials; and (3) to evaluate treatment effectiveness by combining and complementing face-to-face visits. Although further studies are needed, it may be used to screen children and their parents who are unsure whether to see a specialist and to provide assessment support for clinics without developmental testing capabilities.

Nevertheless, a careful balance must be maintained, as direct in-person assessment provides advantages such as observing nonverbal cues and behaviors, which may be important for a comprehensive understanding of the child’s condition.

### Limitations

This study had several limitations. First, it was limited to children who had already been diagnosed and had received medical care and treatment. Therefore, these results do not apply to undiagnosed neurotypes. This study aimed to explore whether remote assessment tools could be helpful when children with developmental issues and their caregivers seek medical assistance. Therefore, the study design did not encompass typically developing children. Nonetheless, ICC, which usually tends to exhibit higher values when there is a diverse range of patient scores, manifested a relatively high degree of agreement, specifically in the group of children affected in this study. This can be interpreted as endorsing the validity of remote assessment procedures in this context. Second, although our study effectively indicated the potential for high-accuracy remote assessments, this does not necessarily guarantee a remote diagnosis. However, considering the frequent use of the ADHD-RS-IV in the diagnostic process for ADHD, the fact that we have demonstrated its robustness in the context of remote assessment may suggest its utility for future diagnoses. Even if remote ADHD-RS-IV assessments do not replace diagnosis during the first visit, they can be used to identify individuals who should be prioritized for early assessment by conducting severity evaluations and triages, thus expediting their initial consultation. To compensate for the weaknesses of this study, future studies should focus on examining the congruence between severity assessments and diagnoses conducted remotely in comparison with in-person evaluations, in addition to evaluating the efficacy of remote methods across diverse subpopulations.

### Comparison With Previous Work

There have been few studies comparing ADHD assessment scales in face-to-face and remote settings. In previous research in the field of neurodevelopmental disorders, there is a report on the usability and reliability of the Autism Diagnostic Observation Schedule conducted face-to-face and remotely with 23 adults with ASD [16]. The ICC between face-to-face and remote was high at 0.92. In their report, technicians were present in the room during the remote assessment to provide assistance with technical operations, whereas in this study, we made it possible for participants to operate the equipment themselves at home without the help of technicians. Additionally, in that study, the same examiner conducted the tests in both face-to-face and remote settings for all cases. In contrast, in our study, different examiners conducted the tests in face-to-face and remote settings. Although it is difficult to compare because the disorder and assessment tools are different, considering these factors, the ICC value of our results is comparable, indicating a new finding in this field.

### Conclusions

The results of this study showed that developmental assessments can be conducted with the same level of accuracy using remote tools as compared to face-to-face assessments. This means that even medical institutions where specialized assessments are not available, as well as health care centers, can benefit from these assessments, thereby improving the convenience for children who require early detection and intervention. Future research is needed to investigate the consistency of remote assessments and diagnoses compared with the initial face-to-face examination as well as the effectiveness of remote examinations in various subpopulations.

## Abbreviations

ADHD: attention-deficit/hyperactivity disorder
ADHD-RS-IV: Attention-deficit/Hyperactivity Disorder Rating Scale-IV
ASD: autism spectrum disorder
DSM-5: Diagnostic and Statistical Manual of Mental Disorders, Fifth Edition
ICC: intraclass correlation coefficient
